# Prosthetic Heart Valves: More than Half a Century of Innovation—An Overview

**DOI:** 10.3390/jcm14103499

**Published:** 2025-05-16

**Authors:** Asna Tabassum, Katherine G. Phillips, Fadi Hage, Ali Hage

**Affiliations:** 1 Department of Cardiothoracic Surgery, NYU Langone Medical Center, New York, NY 10016, USA; asna.tabassum@nyulangone.org (A.T.); katherine.phillips@nyulangone.org (K.G.P.); 2 Lankenau Medical Center, Wynnewood, PA 19096, USA

**Keywords:** valvular heart disease, prosthetic valves, mechanical valves, valve replacement, valve innovation, transcatheter valve replacement, cardiac surgery

## Abstract

Since Dr. Charles Hufnagel introduced the first ball-in-cage valve prosthesis in 1952 to treat a patient with aortic regurgitation, the field of valvular heart disease has undergone remarkable evolution in both prosthetic valve development and patient management. Over the past 73 years, a wide range of valvular prostheses have been developed, each offering distinct advantages in terms of durability, thrombogenicity, and hemodynamics. This review aims to provide a detailed discussion of commonly known and used valvular heart prostheses, along with a review of newer endovascular prostheses. As ongoing research and innovation continue to shape the field, we can expect further improvements in hemodynamics, clinical outcomes, cost, ease of operation, and patient quality of life.

## 1. Background

Valvular heart disease typically presents as stenosis, regurgitation, or a combination of both, with symptoms including chest pain, palpitations, shortness of breath, fatigue, lightheadedness, fainting, or swollen limbs. These symptoms can severely impact daily functioning and significantly reduce quality of life. The leading global cause of valvular pathology is rheumatic heart disease (RHD), often involving both the mitral and aortic valves and presenting initially with regurgitation before potentially progressing to stenosis. In 2019, an estimated 40.5 million people were affected by RHD [1]. In contrast, the most common valvular disorder in Europe and North America is aortic valve stenotic disease (ASVD), a progressive calcification of a trileaflet or congenitally bicuspid valve. ASVD prevalence increases with age, affecting approximately 0.2% of individuals aged 50–59 and up to 9.8% of those aged 80–89 years [2]. Valvular heart disease remains a significant global health burden, with the prevalence of both RHD and ASVD as well as other pathologies continuing to rise.

Surgical interventions aim to correct valvular dysfunctions, alleviating symptoms and restoring physiological hemodynamics. Attempts at valve replacement date back to the 1920s, and it was in 1952 that Dr. Charles Hufnagel placed the first ball-in-cage valve prosthesis in the descending aorta of a patient with aortic regurgitation [3]. Since then, remarkable advancements have been made in valve replacement technology. Valvular prostheses are now broadly classified as mechanical or biologic valves. Mechanical valves include early designs such as the ball-in-cage, as well as more refined configurations like monoleaflet (tilting disc) and bileaflet valves. Biologic valves, typically composed of either porcine or bovine tissue, can be further categorized as stented, stentless, or sutureless depending on their structural support and implantation techniques. In addition to these, other options such as homografts (human donor valves) and composite valve grafts (valves integrated into vascular conduits, often used in aortic root replacement) are used in select clinical scenarios. A more recent addition to the cardiac valve replacement repertoire is the use of minimally invasive valve replacements, such as in the forms of transcatheter aortic valve replacement (TAVR) and transcatheter mitral valve replacement (TMVR), as well as tissue-engineered valves. All of these comprise the armamentarium of the cardiac surgeon when selecting their approach to perform a valve replacement. The goal of this review is to describe the major categories of valvular prostheses, their historical and technological development, and their uses in conventional and contemporary cardiac surgery.

## 2. Mechanical Prostheses

Mechanical valves, generally composed of a metal or carbon alloy, offer greater durability than biologic valves due to their resistance to degeneration over time. However, these valves are prothrombogenic and therefore require long-term anticoagulation with vitamin K antagonists. The target International Normalized Ratio (INR) differs according to the position of the mechanical valve, ranging between 2 and 3 for the aortic position and between 2.5 and 3.5 for the mitral position [4]. Risk of major embolism without anticoagulation, with antiplatelet therapy, or with warfarin in mechanical heart valves was found to be 4 per 100 patient-years, 2.2 per 100 patient-years, and 1 per 100 patient-years, respectively, when implanted in the mitral or aortic position [4]. Warfarin remains the standard of care in spite of newer, more patient-friendly direct oral anticoagulants (DOACs), such as dabigatran and apixaban, both of which failed clinical trials for their inability to effectively prevent mechanical valve thrombosis; this is possibly due to their different mechanisms of action in inhibiting the coagulation cascade [5,6]. In the context of warfarin, adequate control of the INR is necessary to reduce the risk of thromboembolism when the INR is subtherapeutic or the risk of bleeding when the INR is supratherapeutic. Consequently, mechanical valves tend to have more frequent bleeding complications than biologic valves [7]. Usage of the mechanical heart valve has thus declined in recent decades due to patient aversion to long-term anticoagulation therapy [8]. Still, mechanical valves remain an attractive option in certain use cases, such as in younger patients with longer life expectancies, those with pre-existing indications for anticoagulation such as atrial fibrillation, or those with small annuli or aortic roots where modern mechanical valves may offer superior hemodynamic performance due to larger effective orifice areas, lower transvalvular gradients, and better flow dynamics compared to biologic options [8,9].

### 2.1. Ball-in-Cage (Starr–Edwards)

**Figure 1:**
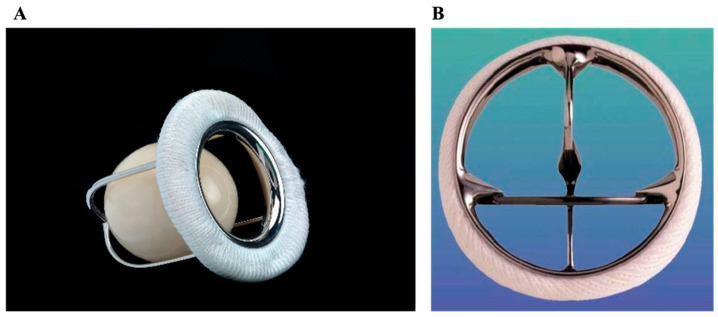
Early mechanical valves. (**A**) Ball-in-cage (Starr–Edwards); (**B**) Monoleaflet Medtronic-Hall tilting disc.

Designed in the 1960s as one of the earliest prosthetic valves invented, the Starr–Edwards valve (Figure 1A) [10] consisted of a ring and three bent metallic arcs forming a cage encasing a silicone ball [11]. It was placed in the aortic or mitral position. When used in the aortic position, this prosthesis was placed at the level of the aortic annulus, with the apex of its cage facing the ascending aorta. In diastole, the ball sat in its ring and blocked the aortic orifice to prevent regurgitation; however, in systole, the left ventricle had to exert a significant force to project and displace the ball in order to eject the blood into the systemic circulation [12]. Similarly, when in the mitral position, the ball was displaced in diastole to allow for ventricular filling; pressure in the ventricle during systole then pushed the ball to occlude the ring, preventing mitral regurgitation.

During its peak time of use, its contemporary in the field of valve replacements was the glutaraldehyde-fixed xenograft valves (an early version of today’s biologic valves), which tended to demonstrate similar risk of mortality [13]. The ball-in-cage prosthesis had less satisfactory hemodynamic properties and provided one of the smallest effective orifice areas compared to more modern valve options. As a result, common postoperative complications included increased pressure gradients, pulmonary hypertension, and acute left ventricular dysfunction [14]. It also presented a higher thromboembolic risk than other mechanical valves [15]. For these reasons, the ball-in-cage valve declined in use and was formally discontinued in 2007 [16]. However, these valves demonstrated remarkable durability, with many reports of function lasting over 40 years [17]. While no longer surgically implanted, they remain relevant as they can still be found in patients today.

### 2.2. Monoleaflet

A monoleaflet valve consists of a mobile tilting disc positioned in the horizontal plane of a ring. The disc tilts approximately 60° to 80° from its neutral position, forming two orifices [11]. This valve can be used in the aortic or mitral position. The older generation Bjork–Shiley valve, developed in the late 1960s, posed a risk of fracture of the hinge that held the disc inside of the ring, and it was therefore withdrawn from the market in 1986 after several reports of disc dislodgement [12]. The next-generation Medtronic-Hall tilting disc (Figure 1B) [18] was introduced in the late 1970s, providing excellent hemodynamic performance in terms of turbulence and transvalvular pressure gradient [19]. Animal studies demonstrated a lower risk of thrombosis in monoleaflet valves versus bileaflet valves, though this difference in human studies was not demonstrated [20].

### 2.3. Bileaflet

This valve is made up of two semilunar discs placed horizontally inside a ring and held in place by two separate hinges. The two discs tilt at approximately 75° to 90° from their neutral positions, forming one central and two lateral orifices [11]. This valve thus offers a greater effective orifice area for the ejection of blood [12]. However, the increased effective orifice area due to this hinged model comes at the price of increased flow stagnation and shear stress, which contribute to a higher thrombotic potential when compared to monoleaflet valves [21]. Earlier studies found that there was a survival advantage to patients aged 50 to 70 years in using a bileaflet mechanical valve versus a stented biologic valve [22].

#### 2.3.1. St. Jude Medical (SJM) and Sorin (CarboMedics–CM)

**Figure 2:**
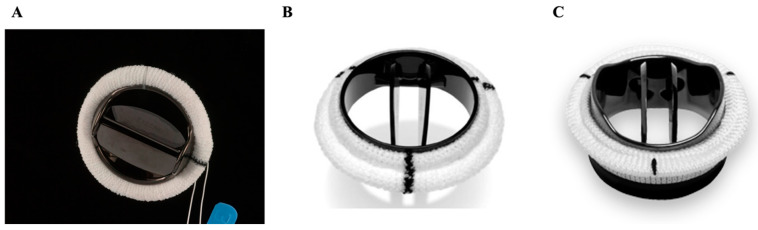
Bileaflet mechanical valves. (**A**) St. Jude Medical (SJM). (**B**) ATS Medical. (**C**) On-X.

These two valves are among the most commonly used bileaflet mechanical valves and can be placed in the aortic, mitral, or tricuspid positions. They are made of pyrolytic carbon, giving them excellent durability. The SJM (Figure 2A) [23], introduced in 1977, has an opening angle of 85°; the CM was introduced in 1986 and has a smaller opening angle of 78° with the intent of reducing regurgitation. The CM’s most distinguishing feature is its capacity for rotation within the sewing ring [24]. In terms of performance, both valves demonstrate similar hemodynamic outcomes and mortality risk in the aortic position, although the CM valve may present a slightly higher thromboembolic risk when used in the mitral position [25,26,27].

#### 2.3.2. ATS Medical

The ATS Medical valve (Figure 2B) [28] is made of pyrolytic carbon, which gives it excellent durability and integrity, and it can be used in the aortic or mitral position. The full opening angle of 85° and opening dynamics of its pivot reduce blood stagnation around the valve, offering it one of the lowest rates of thromboembolic events among mechanical valves [29]. In addition, between its advent in 1977 and 2001, over 50,000 implants of this valve were performed worldwide; during that time, not a single postoperative structural failure was reported [30]. It has good transvalvular pressure gradients, except for its two small sizes with mid-term comparable outcomes to the SJM valve [29,31].

#### 2.3.3. On-X

On-X valves (Figure 2C) [32] can be placed in the aortic or mitral position. The pyrolytic carbon used to make this valve does not contain a silicone alloy, which gives it greater strength and malleability and reduces its thrombogenicity. An interesting feature of the On-X valve is its potential to be used with a lower INR target between 1.5 and 2.0 in the aortic position for patients with no thromboembolic risk factors, making it a good choice for those at high risk of bleeding. The valve has a full opening angle of 90° and excellent transvalvular pressure gradients [33]. Rates of thromboembolism and hemorrhage with On-X mechanical prosthesis compared favorably with past published results of other bileaflet mechanical heart valves in both the aortic and mitral positions [34]. Long-term clinical outcomes are also seen favorably in this valve, with low rates of replacement, reoperation, thromboembolism, and mortality [35].

## 3. Biologic Prostheses

Biologic valves consist of tissue leaflets and thus do not require long-term anticoagulation; this, among other factors, contributed to their dramatic rise in popularity over mechanical prostheses since the late 1990s. However, mechanical and chemical factors together cause structural valve deterioration (SVD) in the form of calcification and disrupted fiber bundles. This degeneration and consequently shorter durability mean approximately 10–30% of biologic valves will require replacement after 10 to 15 years from their implantation. Thus, these valves are rarely used in patients under the age of 50 years unless anticoagulation is refused or contraindicated, such as for women contemplating pregnancy, or a Ross procedure (requiring a homograft) cannot be completed [10,12]. Biologic valves have additionally been demonstrated to have a less significant risk of complications in end-stage renal patients using dialysis compared to mechanical valves—partially because anticoagulation is uniquely challenging in dialysis patients—without significant evidence of accelerated degeneration of the biologic valves in this patient population [36]. Currently, with the significant expansion of percutaneous valve replacements, it is believed that the use of biologic valves may increase in younger patients, given the possibility of the ‘valve-in-valve’ technique. This technique prevents the need for subsequent open/conventional redo surgery, and the current data demonstrates favorable outcomes for this method, with the majority of patients showing favorable survival on short- and mid-term follow-up [37].

### 3.1. Stented

Stented valves are made of a frame/stent that surrounds the biologic valve. The stent provides an annular frame that is held up by a support rail, which contains an outflow and inflow side. The stent itself can be flexible or rigid. These flexible stents are believed to decrease the tensile stress at the commissures of the valves in comparison to rigid stents [38]. Compared to mechanical valves, stented biologic valves generally have smaller effective orifice areas [39]. The two most commonly used types are the porcine and the bovine pericardial valves.

#### 3.1.1. Stented Porcine

**Figure 3:**
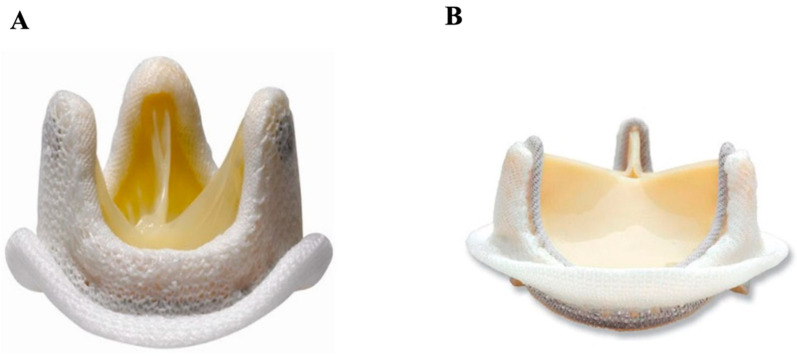
Stented biologic valve. (**A**) Medtronic Mosaic porcine valve; (**B**) Edwards PERIMOUNT pericardial bovine valve.

The stented porcine biologic valve (Figure 3A) [40] can be made either from a single intact valve taken from the pig or reconstituted from several valves. These valves were initially preserved in a formalin solution; however, many of these stented porcine valves would fail within four years of insertion due to structural failure [41]. Now preserved in glutaraldehyde, this valve can be used in the aortic, mitral, or tricuspid position and has much better long-term clinical outcomes [12]. In general, the long-term hemodynamic and clinical outcomes of stented porcine biologic valves are satisfactory, with freedom from adverse events at 8.5 years being over 95% for thrombosis, structural and non-structural dysfunction, hemorrhage, endocarditis, and need for reoperation [42].

#### 3.1.2. Stented Pericardial Bovine

The stented pericardial bovine valve (Figure 3B) [43] is made of a bovine pericardium folded in the form of three cusps, which are then reinforced by a frame or stent [12]. An in-vitro study comparing various stented tissue valves demonstrated that bovine pericardial valves were superior to porcine valves in terms of residual transvalvular gradient and valvular orifice area [44]. In addition, bovine pericardial valves had less obstruction compared to stented porcine valves, though similar quality of life and mortality were demonstrated [45]. At 5, 10, and 15 years of follow-up, freedom from reoperation was 99%, 94%, and 77%, respectively [46]. In the aortic position, there appears to be no significant difference in long-term survival or reoperation between stented porcine and bovine prostheses, regardless of valve size or patient age [47].

### 3.2. Stentless

Stentless biologic valves consist of a tubular body with a flow passageway and a suture ring with an annular cord that is fixable to the patient’s aortic ring. Without the stent and the sewing ring, there is less obstruction of blood flow. Thus, this type of biologic valve was created to improve durability and hemodynamic performance compared to other valvular prostheses. Long-term studies have demonstrated a significant reduction in transvalvular gradients and a larger valvular orifice with this type of valve in comparison with stented biologic valves. However, studies show similar left ventricular remodeling and patient functional status between both types of valves [48]. In addition, the surgical implantation of stentless biologic valves demands more technical complexity with longer bypass and cross-clamp times [49]. Overall, however, this prosthetic option offers excellent outcomes through 10 years of follow-up, regardless of its implantation [50].

#### 3.2.1. Stentless Pericardial Bovine

**Figure 4:**
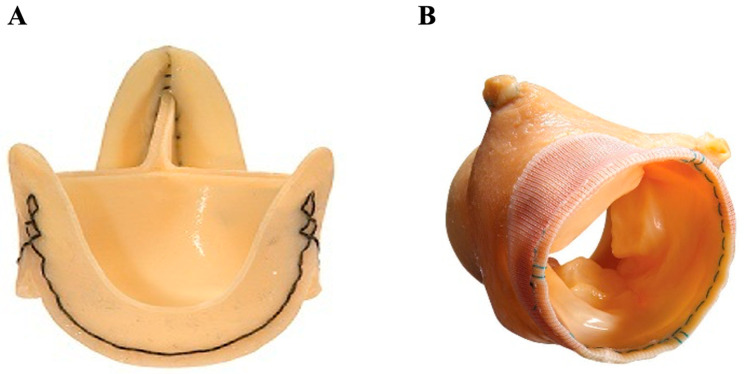
Stentless bioprosthetics. (**A**) Sorin Freedom SOLO (pericardial bovine); (**B**) Medtronic Freestyle (porcine).

The SORIN Freedom Solo (Figure 4A) [51] is a stentless pericardial bovine aortic prosthesis implanted in the supra-annular position. It is relatively easy to implant in comparison to other stentless valves, requiring only a single suture line, and shows excellent hemodynamic performance on medium-term follow-up [52]. The Solo stentless valve, when compared with stented biologic or mechanical valves, demonstrated more rapid regression of left ventricular hypertrophy one year post-surgery [53]. A higher risk of thrombocytopenia was observed with this valve when compared with the Carpentier–Edwards Perimount bovine pericardial valve [54]. In a large, multicenter European study, the long-term durability and hemodynamics of the Freedom Solo stentless valve were demonstrated to be excellent [55].

#### 3.2.2. Stentless Porcine

Medtronic’s stentless Freestyle valve (Figure 4B) [56] consists of a porcine aortic root, used for replacement of the aortic valve and the aortic root simultaneously. It was first developed by Tirone E. David and colleagues in a sheep model, with preliminary clinical findings reported in 1990 [57]. This biologic valve demonstrates excellent hemodynamic performance on medium-term follow-up, with good transvalvular gradients and valvular orifice area, in addition to left ventricular remodeling [58]. A European study corroborates these findings, demonstrating larger effective orifice area, lower pressure gradients, better left ventricular remodeling, as well as lower complication and reoperation rates, in stentless porcine aortic valves versus early stented models [59]. However, with modern improvements in stented valve design, more recent research demonstrated similar hemodynamics in stentless versus modern stented valves [60]. Studies have additionally found that reoperation of stentless aortic valve replacements is associated with increased risk of death due to its challenging procedural nature [61].

### 3.3. Sutureless

**Figure 5:**
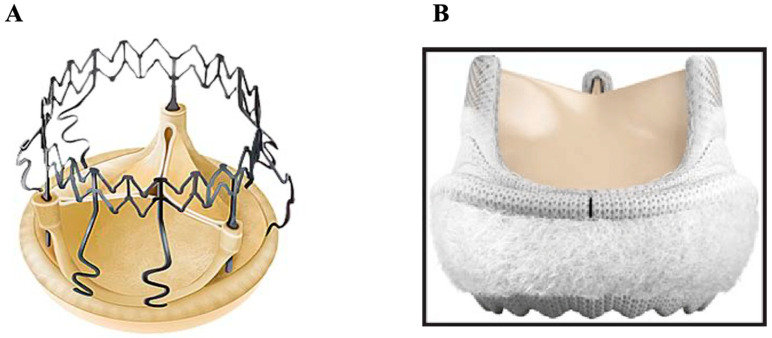
Sutureless biologic valves (bovine pericardium). (**A**) Sorin Perceval S; (**B**) Edwards Intuity Elite.

‘Sutureless’ valvular prostheses are relatively newer, stented aortic prostheses rapidly deployed in their position with the added advantage of requiring a smaller right anterior minithoracotomy rather than a typical median sternotomy. The two most popular sutureless biologic valves are the Perceval S and Intuity Elite (Figure 5A,B) [62,63], both of which are made of a bovine pericardium mounted under a flexible metallic frame that is deployed at the level of the aortic root. The former requires no sutures, while the latter requires only three. Sutureless valves are associated with reduced aortic cross-clamp and cardiopulmonary bypass times—in the case of the Perceval S, an average aortic cross-clamp as low as 18 ± 6 min—potentially reducing short-term mortality and morbidity [64]. Comparable hemodynamics and clinical outcomes were observed between the Perceval S and Intuity Elite, though the Intuity Elite was associated with lower transaortic peak and mean gradients as well as higher effective orifice area [65,66]. When compared to other less invasive approaches such as TAVR, it is hypothesized that because of the excision of the calcified aortic valve and implantation of the sutureless valve under direct visualization, the risk of paravalvular regurgitation should be minimized [67]. At a mean follow-up of 18.9 months, one propensity-match analysis found a significant difference in survival between sutureless (97.3%) and transcatheter aortic valve replacement (TAVR) (86.5%) [68]. However, several studies have reported a higher incidence of heart block requiring permanent pacemakers with this type of prosthesis, as compared to a standard aortic valve replacement [69].

## 4. Other Prostheses

### 4.1. Homograft

**Figure 6:**
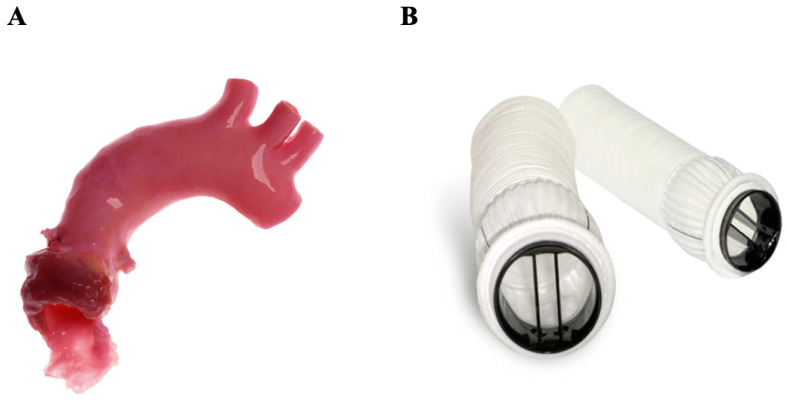
Grafted prostheses. (**A**) Aortic valve homograft conduit; (**B**) On-X ascending aortic prosthesis—a composite valve.

The replacement of a valve by another human valve was first introduced in 1956. The most well-known example is the Ross procedure, a highly complex operation in which the native pulmonary valve is used as a substitute for the pathological aortic valve and a homograft is implanted in the pulmonary position (Figure 6A) [70]. Degeneration of the homograft occurs faster in the aortic position than at the pulmonary valve position due to the patient’s immunologic response to the graft and increased shear stress, which is why the homograft is placed at the pulmonary position and the native pulmonary valve placed at the aortic position. In general, candidates for aortic homograft replacement are commonly patients with endocarditis, women contemplating pregnancy (with higher thromboembolic risk), and young patients presenting with congenital valvular disease. The homograft provides similar transvalvular hemodynamics and long-term survival to those of biologic valves [71]. Clear evidence from large longitudinal studies demonstrated freedom from reoperation at 15 years in 94% of patients over the age of 60 [72]. The major limitations for this procedure have been the limited availability of homografts and the substantially increased technical and surgical difficulty of the operation [71].

### 4.2. Composite Valve (On-X Ascending Aortic Prosthesis)

A composite aortic valve replacement (Figure 6B) [73] uses a synthetic polyester graft mounted on a mechanical On-X aortic valve. When implanted, typically as part of the gold standard Bentall procedure, it allows for the replacement of the aortic valve, the Valsalva sinuses, and part of the pathological ascending aorta. Etiologies requiring this type of intervention include annuloaortic ectasia and Marfan syndrome, as well as aortic dissections extending into the aortic root. This procedure has a higher level of technical difficulty and is associated with an increased risk of early death when compared to isolated aortic valve replacement [74].

### 4.3. Transcatheter Aortic Valve Replacement (TAVR)

**Figure 7:**
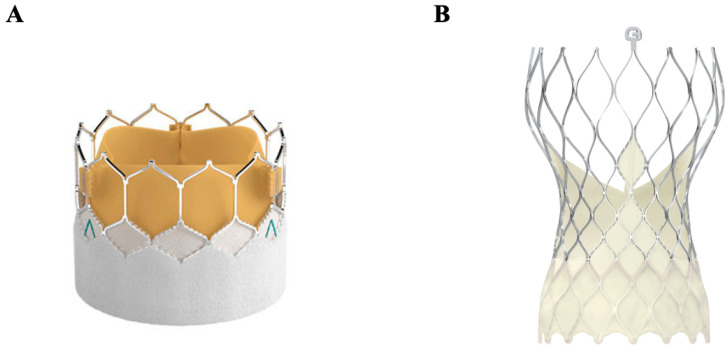
Transcatheter aortic valves. (**A**) Edwards SAPIEN 3 balloon-expandable valve. (**B**) Medtronic Evolut self-expanding nitinol valve.

Transcatheter-based therapies were first introduced in 2000, responding to a need for intervention in high-risk and inoperable patients with valvular disease. Delivered via transapical, transcarotid, direct aortic, and subclavian approaches, although most commonly transfemorally, transcatheter aortic biologic valves are mounted in a metallic frame and positioned at the aortic annulus. Mechanisms of deployment differ, involving either a balloon-expandable valve such as in the Edwards SAPIEN model (Figure 7A) [75] or a self-expanding nitinol valve such as in Medtronic’s CoreValve and Evolut (Figure 7B) [76]. Models [12] While typically performed under general anesthesia, TAVRs can alternatively be performed under local anesthesia, allowing for decreased time of the procedure and a shorter hospital stay [77]. The safety of TAVR was first reported in 2010 and showed symptomatic improvement in surgically inoperable patients [78], who comprise approximately 30% of severe aortic stenosis patients [79]. Multiple subsequent clinical trials have been conducted and expanded the indication of this novel technology to patients at high [80], intermediate [81], and, most recently, low surgical risk [82]. In addition, given the possibility of performing “valve-in-valve” implantations, it has increased in popularity among younger patients in whom the risk of valvular degeneration is high and among older patients with biologic valves requiring reoperation. Between 2013 and 2017, TAVR volumes increased 30–49% per year in the United States [83]. However, TAVRs face obstacles with certain unfavorable anatomies, such as the extremes of annulus size, low coronary height (associated with a higher risk of coronary artery obstruction), and iliofemoral vascular disease, calcification, or tortuosity precluding femoral access [84]. While bicuspid aortic valve patients represent a growing population of TAVRs, long-term durability remains unknown in this group [84] and in all low-risk groups. Additionally, in comparison to surgical implantations, TAVRs have been associated with heart blocks requiring pacemaker implantation, paravalvular regurgitation, and stroke; while having improved over the last decade, these remain persistent concerns [85]. Pure aortic regurgitation has also been a challenge for TAVR because of the difficulty of anchoring the valve in a non-calcified landing zone. Novel techniques are being proposed in this patient population, consisting of implanting an uncovered stent in the landing zone, followed by the deployment of TAVR within this “prestented” landing zone [86].

### 4.4. Transcatheter Mitral Valve Replacement (TMVR)

TMVR has emerged as an alternative option for patients with severe mitral regurgitation (MR) with a prohibitive or high surgical risk. TMVR is most frequently performed via transapical access. Early results have shown excellent transvalvular gradients and very low rates of residual moderate MR. Despite these promising hemodynamic outcomes alongside representing a minimally invasive option, TMVR does not have the same early success as TAVR; periprocedural and 30-day mortality are high, with a rate of all-cause mortality around 28% at a mean follow-up of 10 months [87]. The preferred intervention in mitral valve disease continues to favor repair over replacement, though TMVR may serve as a useful alternative to surgical replacement when a replacement is warranted in a high-risk patient. One innovative use of TMVR has been in high-risk patients requiring reoperation for mitral valve replacement. A recent meta-analysis looked at TMVR valve-in-valve or valve-in-ring in high-risk redo mitral valve replacement and found the method to be safe and effective [88].

### 4.5. Transcatheter Pulmonary Valve Replacement (TPVR) and Transcatheter Tricuspid Valve Replacement (TTVR)

The use of transcatheter valve replacement in the pulmonary and tricuspid positions has primarily been studied in patients with congenital heart disease (CHD). As a result, transcatheter pulmonary valve replacement (TPVR) was developed; the first being the Melody Transcatheter Pulmonary Valve, consisting of a bovine jugular vein graft sewn to a metal stent, approved in 2015 [89]. A multicenter study in 2018 presented the use of the Melody Valve for atrioventricular valve replacement in pediatric patients and demonstrated it to be a suitable option given its beneficial short-term function and ability to be later enlarged using a catheter approach as the child ages [90]. There remains a high risk for mortality and valve deterioration in these patients post-operatively, and thus continued development of this technique is certainly necessary [90]. Another valve, the SAPIEN XT, approved for use in 2016, is a tri-leaflet bovine pericardial valve sewn to a steel stent originally designed for the aortic position but can also be used in both the pulmonary and tricuspid positions [89]. Like the Melody Valve, the SAPIEN XT valve has also demonstrated effective relief from regurgitation and stenosis in the pulmonary position in pediatric patients, though it has been associated with complications such as stent fracture and endocarditis [91]. TTVR has also been briefly studied for valve-in-valve or valve-in-ring reoperations with safe and accepted outcomes in congenital heart disease patients, although there has yet to be a comparative study to traditional surgical management [89].

### 4.6. Tissue-Engineered Heart Valves

An area of contemporary research is the development of tissue-engineered heart valves such as decellularized homografts. This is accomplished by reseeding a decellularized, non-fixed valve scaffold with autologous cells from the eventual recipient. This offers two theoretical advantages: functionally, it enables the valve to better replicate native biological valve performance, and longitudinally, it may reduce the autoimmune response and slow down graft degeneration. Additionally, in children, tissue-engineered heart valves have demonstrated the ability to increase in diameter in accordance with somatic growth of the annulus [92]—an especially valuable property, as long-term durability is of high priority in young patients. A 2016 study examined the 10-year follow-up of decellularized fresh pulmonary homografts when used for pulmonary valve replacement in congenital heart disease and found a 100% freedom from explantation, in comparison to 84.2% for cryopreserved homografts and 84.2% for bovine jugular vein conduits [93]. A 2019 study later examined the use of decellularized homografts in five pediatric congenital heart disease patients requiring double semilunar valve replacement, showing excellent valve function at a median follow-up of 31 months [94]. Though early in development, the use of tissue-engineered homografts for valve replacements could be revolutionary, with promising outcomes in preclinical and clinical studies [95]. A summarized overview of the key characteristics, indications, advantages, and disadvantages of the discussed valve types is provided in Table 1.

## 5. Conclusions

Since the birth of cardiac surgery, there has been constant innovation in both the surgical techniques and valvular prostheses available to treat patients with valvular heart disease. In 1960, Starr and Edwards achieved the first successful in situ heart valve implantation in a human, marking the beginning of a transformative, life-saving procedure now performed throughout the world. What began with the implantation of the “ball-in-cage” prosthesis in the descending thoracic aorta has, over decades, evolved into a modern transcatheter valve implantation through a small, almost invisible incision in the groin. Quality-of-life metrics, including physical and mental health, demonstrate significant improvements for patients who choose to undergo valve replacements for their valvular disease. This progress has been driven by the desire of cardiac surgeons to provide the best care for their patients with valvular disease, a condition that remains a leading cause of cardiovascular morbidity and mortality globally. As the burden of this disease is projected to grow, ongoing research and innovation will continue, aiming to improve hemodynamics, clinical outcomes, cost, ease of operation, and patient quality of life.

## Figures and Tables

**Table 1:** Summary of valves by type and key characteristics.

Valve Type	Features	Common Indications	Advantages	Disadvantages
Mechanical	Durable metal/carbons; designed for lifetime use	Younger patients; pre-existing anticoagulation needs (e.g., atrial fibrillation)	Superior durability; effective in small anatomy	Lifelong warfarin anticoagulation (target INR 2.0–3.0 in aortic, 2.5–3.5 in mitral)
Ball-in-cage (Starr–Edwards)	Earliest mechanical valve; silicone ball within a metal cage	Historic (no longer used)	Extremely long lifespan (some > 40 years)	Poor hemodynamics; high gradients; high embolic risk; obsolete
Monoleaflet	Single tilting disc design; two asymmetric orifices	Aortic and mitral positions	Better flow than ball-in-cage; simpler design than bileaflet	Older models had fracture risks; lower risk of thrombosis in animal studies
Bileaflet	Two semicircular discs; one central and two lateral orifices, symmetric	Most common mechanical design (aortic, mitral, tricuspid)	Excellent hemodynamics; large orifice; reduced target INR of 1.5–2.0 in On-X	Higher flow stagnation and shear stress than in monoleaflet
Biologic	Tissue-based; typically bovine or porcine	Age > 50, dialysis patients, or anticoagulation contraindications (pregnancy)	No long-term anticoagulation; expanding transcatheter use	Less durable (10–15 years) due to structural valve deterioration
Stented	Biologic valve on a rigid or flexible frame	Aortic, mitral, tricuspid valve replacement	Easier implantation; good mid/long-term safety	Smaller orifice than mechanical; eventual degeneration
Stentless	No stent; tubular body and suture ring; greater native-like flow	Aortic valve replacement	Larger orifice and lower gradients than stented; excellent 10-year data	Longer surgery times; complex implantation; difficult reoperation
Sutureless	Bovine pericardium on metallic frame; minimal or no suturing required	Less invasive aortic replacement (minithoracotomy)	Rapid deployment; reduced clamp time; favorable survival vs. TAVR	Higher heart block and pacemaker rates than standard surgical replacement
Other	Newer innovations	Varied	Varied	Varied
Homograft	Human donor valve; often used in Ross procedure (placed in pulmonary position)	Endocarditis, pregnancy, congenital valve disease	Good hemodynamics; no anticoagulation; excellent long-term outcomes in older adults	Technically challenging; limited availability
Composite	Mechanical valve with synthetic graft; replaces valve and ascending aorta	Bentall procedure; aneurysms; Marfan syndrome; annuloaortic ectasia; dissection into aortic root	Comprehensive repair in one procedure	High technical complexity; increased early mortality risk
Transcatheter (TAVR)	Catheter-delivered biologic valve for aortic position	Aortic stenosis, especially in high-risk groups; valve-in-valve for failed bioprostheses	Minimally invasive; fast recovery; valve-in-valve option	Limited in certain anatomies; associated with heart block, pacemaker, stroke, PVL; uncertain long-term durability
Transcatheter (TMVR/TPVR/TTVR)	Catheter-delivered biologic valves for mitral, pulmonary, or tricuspid positions	High-risk mitral regurgitation; congenital heart disease; pediatric cases	Minimally invasive alternative for reoperations; good initial hemodynamics; valve-in-valve option	Limited data; high mortality in TMVR; complications (fracture, endocarditis) in pediatric TPVR
Tissue-engineered	Decellularized scaffold reseeded with patient’s cells; mimics native valve growth	Pediatric congenital disease; research settings	Somatic growth potential; reduced risk of immune rejection; strong early durability data	Experimental; long-term outcomes not yet confirmed
